# Predicting free edge delamination induced by thermal loading using finite fracture mechanics

**DOI:** 10.1007/s10704-024-00817-z

**Published:** 2025-02-17

**Authors:** Mohammad Burhan, Zahur Ullah, Zafer Kazancı, Giuseppe Catalanotti

**Affiliations:** 1 Advanced Composites Research Group, School of Mechanical and Aerospace Engineering, Queen’s University Belfast, Ashby Building, Belfast, Northern Ireland BT9 5AH UK; 2 Department of Engineering, Durham University, South Road, Durham, DH1 3LE UK; 3 Facoltà Di Ingegneria E Architettura, Università Degli Studi Di Enna Kore, Cittadella Universitaria, 94100 Enna, Italy

**Keywords:** Thermal loading, Finite fracture mechanics (FFM), Energy release rate (ERR), Interlaminar stresses, Semi-elliptical crack, Free edge effect

## Abstract

The material mismatch between the dissimilarly oriented plies within laminated structures induces localised singular interlaminar stresses at free edges, under various loading conditions such as mechanical, moisture, or thermal. These interlaminar stresses lead to premature interlaminar cracking. This study introduces the application of Finite Fracture Mechanics (FFM) for predicting free edge delamination in angle-ply laminates under uniform thermal loading. The current framework assumes nucleation of semi-elliptically shaped crack at the dissimilar interface, resulting in a 3D FFM criterion. For a given material intrinsic properties, e.g. interlaminar fracture toughness and strength, calculation of quantities such as interlaminar stresses and incremental energy release rates are required. These quantities, necessary for the evaluation of the FFM criterion, are determined semi-analytically through expressions derived from dimensional analysis and finite element models. Dimensional analysis facilitates the finding of these quantities only once using non-dimensionalised functions. The resulting non-dimensionalised functions for stresses and energy release rates are not a function of thermal load and ply thickness. This eliminates the requirement to re-solve the underlying boundary value problem for varying loads and ply thicknesses. The accuracy of finite element models is confirmed against results from models available in literature and dimensional analysis is validated against numerical solutions. The 3D FFM system is solved by assuming a homothetic crack extension and is implemented as a standard constrained nonlinear optimisation problem. In addition to the 3D FFM, another model based on the Theory of Critical Distances (TCD) is employed for validation purposes. The predictions from both the 3D FFM and TCD are compared to those from models available in the literature.

## Introduction

Fibre-reinforced polymer composites possess exceptional properties, including superior fatigue life, outstanding corrosive resistance, high specific strength, and stiffness and therefore have been widely utilised in aerospace, marine, automobile, and construction industries (Li et al. 2019, 2006; Guan et al. 2020; Bruggi and Taliercio 2013). The effective properties of the composite laminates can be adjusted based on the orientation of the individual plies. However, this ply orientation mismatch in adjacent layers can induce stress singularities at the laminate free edge and consequently may yield interlaminar fracture under quasi-static, thermal or fatigue loading conditions. This phenomenon, known as free edge effect, is originally introduced by Hayashi (1967). Since no exact solution exists for the free edge effect (Mittelstedt et al. 2022), significant efforts have been made by the scientists during the last fifty years to understand its behaviour. Following the pioneering contributions of Pipes and Pagano (1970), this includes employing semi-analytical (Mittelstedt and Becker 2005; Dölling et al. 2020), numerical (Islam and Prabhakar 2017; Raju and Crews 1981; Ye and Yang 1988), and closed-form approaches (Pagano and Pipes 1971; Kassapoglou and Lagace 1987; Wang and Choi 1982; Mittelstedt and Becker 2007a; Sarvestani and Sarvestani 2012). Besides the above-mentioned methods, a number of review papers exist [see references Mittelstedt et al. (2022), Salamon (1980), Kant and Swaminathan (2000), Mittelstedt and Becker (2007b)].

The fracture criteria used to predict the free edge delamination can be categorised into either stress-based or energy-based. Due to existence of singular stresses at the free edge (Wang and Choi 1982), conventional local strength of materials criteria are always satisfied. Conversely, Linear Elastic Fracture Mechanics (LEFM) requires the presence of a flaw to function, making it impractical for flaw-free structures. Alternatively, non-local strength-based failure criteria, which involves averaging interlaminar stresses within a certain distance from the free edge (referred to as critical length), is viable to overcome issues with singularities. For instance, Kim and Soni (1984), Zhou and Sun (1990), Lagunegrand et al. (2006), and Brewer and Lagace (1988), have utilised this average stress criterion, drawing inspiration from Whitney and Nuismer (1974) and Neuber (1934). Another category comprises fracture mechanics-based criteria, which necessitates the assumption of the existence of an inherent flaw and requires the calculation of interfacial energy release rate. The implementation of this type of criterion can be seen in the works of Wang and Crossman (1980), O’Brien (1982), Rybicki et al. (1977) and Leguillon (1999). These non-local failure criteria are summarised as the Theory of Critical Distances (TCD), a term introduced by Taylor (2008). However, all the aforementioned approaches rely on an unknown empirical length, necessitating its prior determination through experimentation and lacking a clear physical meaning. For this reason, Leguillon (2002) introduced a coupled stress and energy criterion, within the framework of Finite Fracture Mechanics (FFM), which eliminates the requirement of prior assessment of the critical length, relying only on intrinsic material properties such as fracture toughness and strength. FFM has been effectively used in numerous structures containing singular and non-singular stress raisers, such as V-notches (Leguillon 2002; Carpinteri et al. 2008; Sapora et al. 2013), open-hole plates (Weißgraeber et al. 2016a; Camanho et al. 2012; Martin et al. 2012), bolted joints (Catalanotti and Camanho 2013), and transverse cracking in cross-ply laminates (García et al. 2016). It is noted that FFM has also been applied to 3D cases. In 2D cases, a crack is defined by its length and orientation. The nucleation of a 3D crack is notably complicated since it is characterised by an infinite number of variables that describe its shape. Following the work of Leguillon (2014), who used matched asymptotic expansions to extend the FFM coupled criterion to 3D, García et al. (2016) implemented the 3D FFM to transverse cracking of cross-ply laminates, and Doitrand et al. (2017) used it to woven composites to predict strain at which damage initiates. In order to predict the initiation of cracks in aluminium-epoxy specimens under four-point bending, Doitrand and Leguillon (2018a) used interface normal stress isocontours to determine the crack shape, which is dependent on a single variable. Afterwards, they used the same method—where crack’s shape is parameterised by its surface area—to predict crack onset in scarf adhesive joints (Doitrand and Leguillon 2018b). The detailed review papers are provided by Weißgraeber et al. (2016b) and Doitrand et al. (2024) on the theory and application of FFM. In regards to free edge application of laminates under quasi-static loading conditions, Martin et al. (2010), Hebel et al. (2010), Frey et al. (2021a), and Dölling et al. (2020) implemented FFM to predict the free edge delamination in composite laminates. However, all these FFM models for free edge delamination prediction mentioned above are based on the generalised plane strain state. For this reason Burhan et al. (2024a) recently implemented 3D FFM for free edge delamination prediction by considering and asserting the nucleation of semi-elliptically shaped crack from the free edge. The homothetic crack extension is hypothesised and the FFM system is solved for a unique solution through a standard non-linear optimisation technique. A recent critical review on methods to predict free edge delamination can be found in Burhan et al. (2024b).

It is noteworthy that most of the literature addressing the free edge effect focuses on mechanical loading conditions, with relatively fewer studies investigating thermal loading conditions. Herakovich (1976) conducted an investigation on composite laminates with free edges using finite element analysis and noted that thermal stresses can exceed the stresses induced by mechanical loading. Similarly, Wang and Crossman (1977) employed a finite element approach to analyse the free edge behaviour of symmetric laminates under uniform temperature changes, highlighting the existence of singular shear stress distribution at dissimilar interfaces in angle-ply laminates. Wu (1990) developed a quasi-three-dimensional iso-parametric finite element method for nonlinear thermal analysis of symmetric composite laminates. This analysis involved determining thermal stresses induced from thermal cooling followed by the application of uniform applied strain until failure detection using maximum strain failure criterion. Later, Wu (1992) implemented the same procedure to study thermo-mechanical free edge effects in silicon carbide/aluminium alloy laminates. Webber and Morton (1993) further extended the analytical method presented by Kassapoglou and Lagace (1987, 1986) for evaluating interlaminar stresses due to thermal loads. Yin (1997) developed a variational method based on polynomial expansions of stress functions and the principle of complementary energy, providing closed-form solutions of interlaminar stresses in laminates subjected to uniform and linearly varying non-uniform thermal loads. Diaz Diaz et al. (2002) utilised an approximate approach that models the laminate by a superposition of Reissner plates coupled by interlaminar stresses, revealing the absence of singularities even at the free edge. Tahani and Nosier (2003) developed an elasticity formulation for symmetric and unsymmetric laminates, investigating interlaminar stresses in laminates subjected to layer wise temperature distributions. Nguyen and Caron (2009) applied a multi-particle finite element analysis to predict interlaminar stresses in composite laminates under thermal loading conditions. Islam and Prabhakar (2017) developed and implemented a quasi-2D plane strain formulation to predict interlaminar stresses in multi-directional laminates. In the framework of FFM, Dölling et al. (2021) and Frey et al. (2021b) predicted critical thermal loads of laminates with free edges. However, both studies assumed a generalised plane strain state for the laminate models. Considering the potential for significant temperature changes experienced by composite laminates, especially in aerospace applications, and the existence of residual stresses following curing at elevated temperatures, investigating free edge delamination under thermal loading is of considerable importance and should be integrated into laminate design considerations (Frey et al. 2021b).

In the current study, symmetric angle-ply laminates are investigated under uniform thermal loading, using 3D FFM model. A semi-elliptical crack is assumed to nucleate at the dissimilar interface. Non-dimensionalised functions for both interlaminar stresses and energy release rate (ERR) are determined via expressions obtained from dimensional analysis and finite element models. This semi-analytical framework requires determination of stresses and energy release rates only once for a given ply thickness and thermal load, offering significant computational savings. With three unknowns—thermal failure load and the two semi-axes of the semi-elliptical crack—the FFM involves solving two equations, that yields an indeterminate solution. The unique solution is achieved by minimising the thermal load through a standard nonlinear optimisation problem along with the hypothesis of homothetic crack extension. The current 3D FFM failure thermal load predictions are validated against the Theory of Critical Distance (TCD) implemented as a second fracture criterion, as well as against predictions from 2D FFM and Cohesive Zone Model (CZM) available in the literature.

## Semi-analytical framework

**Fig. 1:**
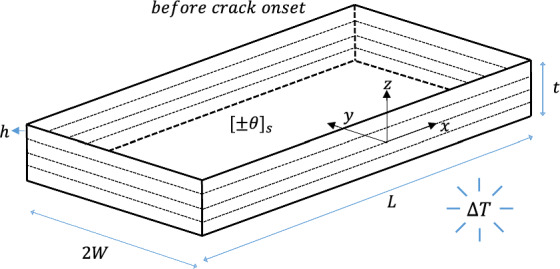
Symmetric uncracked angle-ply laminate subjected to uniform thermal loading

Consider a symmetric four-layer angle-ply laminate with ply orientation $\theta$ subjected to uniform temperature change $\Delta T$, as shown in Fig. 1. Each layer has thickness $h$ and is modelled as homogenous, linear-elastic, and orthotropic material. The laminate has $\left( {L \gg h} \right)$ length, $\left( {2W \ge 16h} \right)$ width, and $t$ thickness, which are maintained throughout the present study. The global Cartesian coordinate system $xyz$ is centred at the free edge of the laminate, located at the ($- \theta / - \theta$) interface. The $x$-axis and $y$-axis are the in-plane directions, while the $z$-axis is through-the-thickness direction of the laminate. All layers are supposed to be perfectly bonded together with a displacement continuity.

**Fig. 2:**
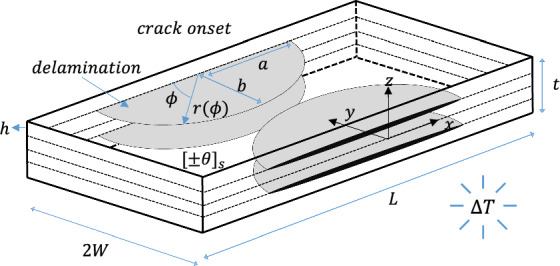
Symmetric cracked angle-ply laminate with four identical semi-elliptically shaped interface delamination located at the $+ \theta / - \theta$ interface under uniform thermal loading

The cracked symmetric angle-ply laminate at the delamination onset is depicted in Fig. 2. In angle-ply laminate under thermal loading, the distribution of interlaminar shear stress at the dissimilar ($\theta / - \theta$) interface exhibits singular behaviour at the free edge (Wang and Crossman 1977). Therefore, delamination failure at this interface primarily occurs due to interlaminar shear stress (Frey et al. 2021b). Consequently, four identical semi-elliptically shaped cracks with semi-axes $a$ and $b$ are supposed to nucleate at the free edge in the dissimilar interfaces, as illustrated in Fig. 2.

Delamination often originates at the interface of the laminate exhibiting free edge effects through the coalescence of inherent flaws, as observed in experimental mechanics, leading to an irregularly shaped delamination front rather than straight front. However, numerical modeling typically simplifies this process by assuming straight cracks. Adopting a semi-elliptical crack shape provides a more realistic representation of delamination extension, even though from a computational mechanics perspective, such cracks may not naturally nucleate from the free edge of the laminate due to uniform stress distribution along the longitudinal direction of the laminate. Nevertheless, the assumption of semi-elliptically shaped crack nucleation from the free edge is meaningful from an experimental mechanics standpoint (Burhan et al. 2024a). This also suggests that predictions of laminate failure stress, assuming semi-elliptical cracks, are as relevant when compared to reference solutions that assume straight cracks, as these straight-crack solutions are when compared with experimental results, where straight crack nucleation is not necessarily observed. Additionally, adopting semi-elliptical crack nucleation offers a basis for understanding 3D crack extension, with the challenge of FFM extension to 3D cases being a significant issue (Weißgraeber et al. 2016b).

### Dimensional analysis

For the general representation of interlaminar stresses and energy release rates, dimensional analysis is performed in this section. The expressions obtained from dimensional analysis for stresses and energy release rates are utilised to determine the corresponding non-dimensional functions. These functions are independent on layer thickness and thermal load. Therefore, interlaminar stresses and energy release rates can be computed for an arbitrary load and layer thickness after non-dimensional functions are known, without requiring re-solution of the boundary value problem. Thus, reducing the significant numerical effort.

Interlaminar stress components of a symmetric angle-ply laminate, for a given material with elastic properties $\left( {E_{1} ,E_{2} , G_{12} ,\upsilon_{12} ,\upsilon_{23} } \right)$, thermal properties ($\alpha_{1} , \alpha_{2}$), geometry $h$, ply orientation $\theta$ and homogenous thermal load $\Delta T$ can be written as:

1 $$ \sigma_{ij} = \sigma_{ij} \left( {\Delta T,y,h, \theta ,E_{1} ,E_{2} , G_{12} ,\upsilon_{12} ,\upsilon_{23} , \alpha_{1} , \alpha_{2} } \right), $$

where $y$ represents the distance from the free edge, and subscripts 1 and 2 correspond to the fibre and transverse directions, respectively.

In this study, the elastic properties are represented by material invariant trace*,* $\Im$, defined in reference Tsai and Melo (2014) and is determined from the 3D elastic stiffness matrix. The Composite Laminate Plate Theory (CLPT) is incorporated to compute the trace-normalised 3D stiffness components for various carbon-fibre reinforced polymer (CFRP) materials. Subsequently, the *Master Ply* is defined using the average values of these components. The individual elastic constants for an arbitrary CFRP material can then be determined by utilising their individual traces and the trace-normalised components of the *Master Ply*.

Following the sequential elimination, for a given layer orientation and material properties (elastic and thermal), Eq. (1) can be expressed in terms of non-dimensionalised groups as:

2 $$ \pi = \pi \left\{ {\frac{{\sigma_{ij} }}{{\Im \alpha_{1} \Delta T}},\frac{y}{h}} \right\}. $$

Thereby, the interlaminar stress components of a symmetric angle-ply laminate for a given material and ply orientation is written as:

3 $$ \sigma_{iz} = \Im \alpha_{1} \Delta T \chi_{iz} \left( \frac{y}{h} \right) \quad i \in \left\{ {x,y,z} \right\}, $$

where $\chi_{iz}$ is a non-dimensional interlaminar stress component.

The procedure to derive an expression for ERR (pertaining to Fig. 2) is analogous to the one utilised above for interlaminar stresses. For a laminate where $$L \gg a,h$$ and $$W \gg b,h$$, the ERR ($$G)$$ depends on the following parameters:

4 $$ G = G\left( {\Delta T,a,b,\phi ,\theta ,h,E_{1} ,E_{2} , G_{12} ,\upsilon_{12} ,\upsilon_{23} , \alpha_{1} , \alpha_{2} } \right). $$

Then following sequential elimination, Eq. (4) is expressed, for a given material properties and ply orientation, in the following dimensionless form:

5 $$ \pi_{G} = \pi_{G} \left( {\frac{G}{{\Im \left( {\alpha_{1} \Delta T} \right)^{2} h}},\alpha ,\beta ,\phi } \right), $$

where $\phi$ represents the polar angle of the point of the contour where ERR is computed (see Fig. 2), $\alpha = a/h,$ and $\beta = b/h$ denote normalised crack semi-axes. Consequently, for a given material system and ply orientation, the ERR along the semi-elliptical crack front at the $\left( { + \theta / - \theta } \right)$ interface in a square symmetric angle-ply laminate is written as

6 $$ G_{i} = \Im \left( {\alpha_{1} \Delta T} \right)^{2} h \psi_{i}^{2} \left( {\alpha ,\beta ,\phi } \right),\quad i \in \left\{ {I,II,III} \right\}, $$

where $\psi_{i}$ represents a non-dimensional correction factor component, and the subscripts ($I,II,III$) denotes modes of fracture.

### Finite fracture mechanics

The FFM, a coupled stress and energy criterion proposed by Leguillon (2002), assumes the spontaneous nucleation of a finite crack size. FFM yields both the unknown finite crack size and the corresponding failure load, if two necessary stress and energy conditions are fulfilled simultaneously. As a result, it eliminates the need for a priori knowledge of an unknown length scale, requiring only intrinsic material properties such as fracture toughness and strength. FFM incorporates the traditional strength-of-materials criterion as a limiting case, applicable when stress singularities are negligible, and fracture mechanics criterion as another limiting case, suitable when strong singularities exist. In this way, FFM bridges the gap between traditional strength-of-materials approaches and fracture mechanics.

The average stress criterion, based on quadratic relation of interlaminar stress components for the free edge delamination and considering the nucleation of a finite semi-elliptically shaped crack, is written as (Burhan et al. 2024a):

7 $$ \frac{1}{A}\mathop \smallint \limits_{ - a}^{a} \mathop \smallint \limits_{0}^{{\frac{b}{a}\sqrt {a^{2} - x^{2} } }} \sqrt {\left( {\frac{{\sigma_{xz} }}{{S_{x} }}} \right)^{2} + \left( {\frac{{\sigma_{yz} }}{{S_{y} }}} \right)^{2} + \left( {\frac{\langle{\sigma_{zz} }\rangle}{{S_{z} }}} \right)^{2} } {\text{d}}y {\text{d}}x \ge 1, $$

where $A = \pi ab/2$ denotes the area of a semi-elliptical crack. $S_{x}$ and $S_{y}$ represent the interlaminar shear strengths for stresses $\sigma_{xz}$ and $\sigma_{yz}$, respectively. $S_{z}$ denotes the interlaminar tensile normal strength. The MacAuley bracket is defined as:

8 $$ \langle\sigma_{zz}\rangle = \left\{ {\begin{array}{*{20}c} {0,} & {\sigma_{zz} < 0} \\ {\sigma_{zz} ,} & {\sigma_{zz} \ge 0}. \\ \end{array} } \right. $$

$\langle\sigma_{zz}\rangle$ only accounts the tensile interlaminar normal stress distribution at the free edge and the influence of compressive stress distribution is not considered.

For thermal load, the interlaminar stress components in Eq. (7) can be expressed from Eq. (3) as:

9 $$ \frac{{2\Im \alpha_{1} \Delta T}}{\pi ab}\mathop \smallint \limits_{ - a}^{a} \mathop \smallint \limits_{0}^{{\frac{b}{a}\sqrt {a^{2} - x^{2} } }} \sqrt {\left( {\frac{{\chi_{xz} }}{{S_{x} }}} \right)^{2} + \left( {\frac{{\chi_{yz} }}{{S_{y} }}} \right)^{2} + \left( {\frac{\langle{\chi_{zz} }\rangle}{{S_{z} }}} \right)^{2} } {\text{d}}y {\text{d}}x \ge 1. $$

Equation (9) is the stress condition for the dissimilar interface in a laminate that is subjected to mixed-mode free edge delamination under uniform thermal loading.

The energy condition for the nucleation of a finite semi-elliptically shaped crack is written as (Burhan et al. 2024a):

10 $$ \overline{G} = \overline{G}_{I} + \overline{G}_{II} + \overline{G}_{III} \ge G_{c} , $$

where $G_{c}$ is mixed-mode interface fracture toughness and $\overline{G}$ represents the total incremental ERR (IERR), which relates the total energy change $\Delta \Pi$ to the emerging finite crack size $\Delta A$ and is written as:

11 $$ \overline{G}_{i} = - \frac{{\Delta \Pi_{i} }}{\Delta A},\quad i \in \left\{ {I,II,III} \right\}. $$

As $\Delta A \to 0$, IERR $\overline{G}$ converges to Griffith’s ERR $G$, and vice-versa. Therefore, $\overline{G}$ can be interpreted as the integral average of $G$:

12 $$ \overline{G}_{i} = \frac{1}{\Delta A}\mathop \smallint \limits_{0}^{\Delta A} G_{i} {\text{d}}A.{ } $$

Combining Eqs. (6), (10) and (12):

13 $$ \overline{G} = \frac{{2\Im (\alpha_{1} \Delta T)^{2} h }}{\pi ab}\mathop \smallint \limits_{ - a}^{a} \mathop \smallint \limits_{0}^{{\frac{b}{a}\sqrt {a^{2} - x^{2} } }} \left( {\psi_{I}^{2} + \psi_{II}^{2} + \psi_{III}^{2} } \right) {\text{d}}y {\text{d}}x \ge G_{c} . $$

Also, the normalised IERR, denoted by ${\Lambda }$, from Eq. (13) can be written as:

14 $$ \Lambda = \frac{{\overline{G}}}{{\Im \left( {\alpha_{1} \Delta T} \right)^{2} h}} = \frac{ 1}{A}\mathop \smallint \limits_{ - a}^{a} \mathop \smallint \limits_{0}^{{\frac{b}{a}\sqrt {a^{2} - x^{2} } }} \left( {\psi_{I}^{2} + \psi_{II}^{2} + \psi_{III}^{2} } \right) {\text{d}}y {\text{d}}x. $$

Equation (13) is the mixed-mode energy condition for nucleation of semi-elliptically shaped crack at a given interface in laminates subjected to thermal loading. Combining both stress and energy criteria, the FFM criterion reads:

15 $$ \left\{ {\begin{array}{*{20}c} {\frac{{2\Im \alpha_{1} \Delta T}}{\pi ab}\mathop \smallint \limits_{ - a}^{a} \mathop \smallint \limits_{0}^{{\frac{b}{a}\sqrt {a^{2} - x^{2} } }} \sqrt {\left( {\frac{{\chi_{xz} }}{{S_{x} }}} \right)^{2} + \left( {\frac{{\chi_{yz} }}{{S_{y} }}} \right)^{2} + \left( {\frac{\langle{\chi_{zz} }\rangle}{{S_{z} }}} \right)^{2} } {\text{d}}y {\text{d}}x \ge 1{ }} \\ { \frac{{2\Im (\alpha_{1} \Delta T)^{2} h }}{\pi ab}\mathop \smallint \limits_{ - a}^{a} \mathop \smallint \limits_{0}^{{\frac{b}{a}\sqrt {a^{2} - x^{2} } }} \left( {\psi_{I}^{2} + \psi_{II}^{2} + \psi_{III}^{2} } \right) {\text{d}}y {\text{d}}x \ge G_{c} } \\ \end{array} .} \right. $$

In the scope of the current paper, since the $\sigma_{xz}$ distribution is predominant in angle-ply laminates at the dissimilar $\left( {\theta / - \theta } \right)$ interfaces, this induces ERR in mixed-mode II/III at the semi-elliptical crack front (Burhan et al. 2024a). Consequently, for angle-ply laminates Eq. (15) reduces to:

16 $$ \left\{ {\begin{array}{*{20}c} {\frac{{2\Im \alpha_{1} \Delta T}}{\pi ab}\mathop \smallint \limits_{ - a}^{a} \mathop \smallint \limits_{0}^{{\frac{b}{a}\sqrt {a^{2} - x^{2} } }} \chi_{xz} {\text{d}}y {\text{d}}x \ge S_{x} { }} \\ { \frac{{2\Im (\alpha_{1} \Delta T)^{2} h }}{\pi ab}\mathop \smallint \limits_{ - a}^{a} \mathop \smallint \limits_{0}^{{\frac{b}{a}\sqrt {a^{2} - x^{2} } }} \left( {\psi_{II}^{2} + \psi_{III}^{2} } \right) {\text{d}}y {\text{d}}x \ge G_{c} } \\ \end{array} .} \right. $$

Equations (16) represents the 3D FFM criterion, which can be utilised for the prediction of the critical thermal load $\Delta T_{f}$ and the associated dimensions of the finite crack $A^{c}$ (expressed as $a^{c}$ and $b^{c}$). For a given interface properties ($S_{x} , G_{c}$), two equations in Eq. (16) have three unknown variables ($\Delta T_{f}$, $a^{c}$*,* $b^{c}$) that corresponds to the critical state and hence infinite many solutions exist. Furthermore, as semi-elliptical crack is assumed to nucleate, Eq. (16) presents difficulties in determining the size and shape of the crack as it extends. The following section is devoted to address these issues associated with the 3D FFM thermal criterion.

### Computation of the critical temperature load

To address the issue of determining crack shape and dimensions during extension, homothetic crack extension, described in Burhan et al. (2024a) is assumed, i.e., the semi-elliptical crack extends in a self-similar manner, thereby maintaining a constant shape throughout the extension, including the aspect ratio $a/b$. Furthermore, there are still infinite crack nucleation configurations possible in the domain where the stress and energy conditions, as a function of two semi-axes, intersect. An additional inequality is introduced, asserting that among all possible fracture thermal load, the critical one is the one that corresponds to minimum. Therefore, these modifications transform the FFM system (16) into a unique solution, achievable via a standard non-linear optimisation problem.

In the numerical solution procedure, $\Delta T_{f}$ and $a^{c}$*,* $b^{c}$, are determined as solutions to the following non-linear constrained standard optimisation problem:

17 $$ \left| {\Delta T_{f} } \right| = \mathop {\text{min}}\limits_{{a,{ }b}} \left[ {{\text{max}}\left( {\frac{A}{{\Im \alpha_{1} s\left( {\frac{{\chi_{xz} }}{{S_{x} }},a,b} \right)}},\frac{1}{{\alpha_{1} }}\sqrt {\frac{{G_{c} A}}{{\Im h g\left( {\psi_{i} ,a,b} \right)}}} } \right)} \right], $$

where $s\left( {\frac{{\chi_{xz} }}{x},a,b} \right)$ and $g\left( {\psi_{i} ,a,b} \right)$ characterise the stress and energy criteria, respectively, as:

18 $$ \begin{aligned} & s\left( {\frac{{\chi_{xz} }}{{S_{x} }},a,b} \right) = \mathop \smallint \limits_{ - a}^{a} \mathop \smallint \limits_{0}^{{\frac{b}{a}\sqrt {a^{2} - x^{2} } }} \frac{{\chi_{xz} }}{{S_{x} }}{\text{d}}y {\text{d}}x \\ & g\left( {\psi_{i} ,a,b} \right) = \mathop \smallint \limits_{ - a}^{a} \mathop \smallint \limits_{0}^{{\frac{b}{a}\sqrt {a^{2} - x^{2} } }} \left( {\psi_{II}^{2} + \psi_{III}^{2} } \right){\text{d}}y {\text{d}}x. \\ \end{aligned} $$

The procedure is subject to a nonlinear equality constraint ensuring that the thermal load causing failure is the same under both conditions:

19 $$ c_{eq} \left( {a,b} \right) = \Delta T\left( {\chi_{xz} ,a,b} \right) - \Delta T\left( {\psi_{i} ,a,b} \right) = 0. $$

The procedure determines the minimum thermal load $\Delta T_{f}$ necessary to nucleate a finite semi-elliptical crack $A^{c}$, with dimensions $a^{c}$ and $b^{c}$, while fulfilling both the stress and energy conditions simultaneously.

### Theory of critical distances

The non-local average stress fracture criterion, that require a priori knowledge of characteristic length, is one of the manifestations of Theory of Critical Distance (TCD). It states that the failure takes place when the average stress distribution over a certain length equals the corresponding material strength. Kim and Soni (1984) implemented first this average stress criterion to free edge delamination in composite laminates, considering interlaminar normal stress distribution, and related the characteristic length with the single ply thickness of the layer.

In this paper, TCD is implemented as second fracture criterion for validation purposes, beside the FFM. The average stress criterion for an interlaminar stress component can be expressed as:

20 $$ \frac{1}{{y_{0} }}\mathop \smallint \limits_{0}^{{y_{0} }} \sigma_{iz} {\text{d}}y = \overline{\sigma }_{iz} \ge S_{i} , $$

where $\overline{\sigma }_{iz}$ represents the average of a stress component, $S_{i}$ denotes the corresponding interlaminar shear strength, and $y_{0}$ indicates the critical length. The interlaminar stress component in Eq. (20) can be reformulated for $\sigma_{xz}$ using Eq. (3) as:

21 $$ \frac{{\Im \alpha_{1} \Delta T}}{{y_{0} }}\mathop \smallint \limits_{0}^{{y_{0} }} \chi_{xz} {\text{d}}y \ge S_{x} . $$

Equation (21) can be written explicitly for $\Delta {\text{T}}$ as:

22 $$ \Delta T \ge \frac{{S_{x} y_{0} }}{{\Im \alpha_{1} \mathop \smallint \nolimits_{0}^{{y_{0} }} \chi_{xz} {\text{d}}y}}. $$

Equation (22) can be used to predict the interlaminar nucleation at the dissimilar $\left( {\theta / - \theta } \right)$ interface in angle-ply laminates for a given $S_{x}$ and $y_{0}$ due to uniform thermal loading. It is noted here that ${\text{y}}_{0}$ is chosen to be equal to a nominal ply thickness, following the assumption by Kim and Soni (1984). Other assumptions have also been proposed, as noted by reference Burhan et al. (2024b): Ye (1988) and Sun and Zhou (1988) used a two-ply thickness, while Brewer and Lagace (1988), Lagunegrand et al. (2006), and Lorriot et al. (2003) suggested determining the best combination of strength and critical length based on experimental data.

### Finite element models

**Fig. 3:**
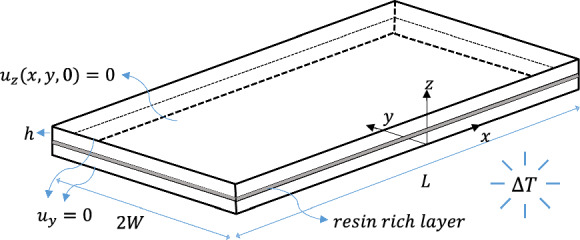
Prescribed and symmetric boundary conditions for a half-uncracked laminate with introduced resin-rich layer at the ($\theta / - \theta$) dissimilar interface

**Fig. 4:**
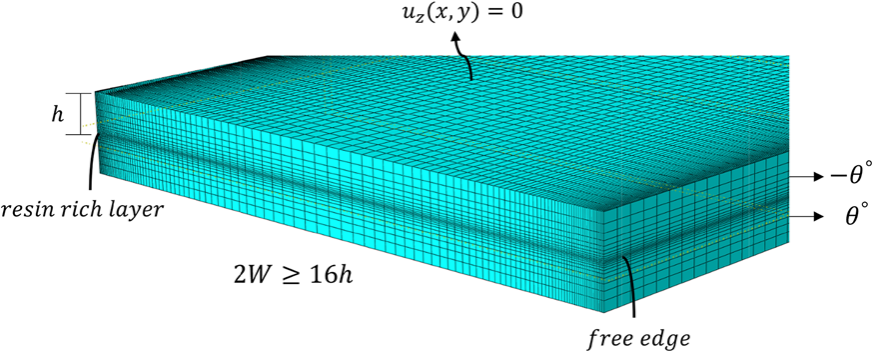
Mesh details in the vicinity of the free edge

Symmetry in angle-ply laminates is only maintained through the thickness in the ($x,y$) plane. This allows the FE modelling of interlaminar stresses to be simplified to half of the structure ($0 < z < 2h$). The resulting two-layer laminate with appropriate boundary conditions are shown in Fig. 3. The computation of interlaminar stresses is facilitated by introducing a thin resin-rich layer at the ($+ \theta / - \theta$) dissimilar interface. It has been found that accurate stress distributions can be obtained at the interface by incorporating a resin-rich layer, which eliminates the need of smoothening and global extrapolation of stresses associated with nodal calculations (Burhan et al. 2024c). The thin resin-rich layer is assumed to have elastic properties with a modulus of elasticity of $E = 3 {\text{GPa}}$ and a Poisson’s ratio $\upsilon = 0.3$. Additionally, the thermal expansion coefficient $\alpha_{p}$ for this resin-rich layer is assumed to be $\alpha_{p} = 22.5 \times 10^{ - 6} \,^{ \circ } {\text{C}}^{ - 1}$, similar to the transverse thermal coefficient of the composite ply. The thickness of the resin-rich layer is considered to be 2% of the ply thickness, with only one through-the-thickness element utilised (Burhan et al. 2024a, 2024c). ${\text{Abaqus}}^{{{\textregistered }}}$ commercial FE software is utilised to compute interlaminar stresses at the Gauss integration points located within this thin layer. The element next to the free edge is situated at $1.25$% of $h$, which is close enough to the condition $y/h = 0$. The 3D FE model in ${\text{Abaqus}}^{{{\textregistered }}}$ utilises 8-node linear brick elements (C3D8R) with reduced integration. Figure 4 illustrates a representative mesh near the free edge.

**Fig. 5:**
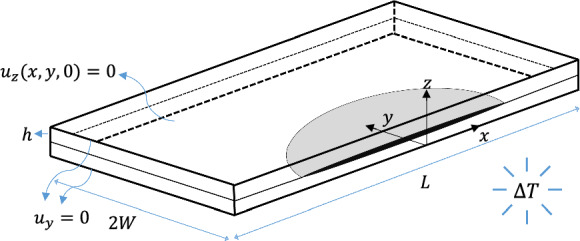
Prescribed and symmetric boundary conditions for a half-cracked laminate, with only one semi-elliptical crack taken account at the ($\theta / - \theta$) dissimilar interface

The appropriate boundary conditions of the half ($$0 < z < 2h)$$ cracked laminate considered for FE modelling are shown in Fig. 5. Within the top half laminate ($$0 < z < 2h)$$, the influence on stress distribution near one crack exerted by the presence of another adjacent crack is not significant. Also, considering that the width $$2W \ge 16h$$ significantly exceeds the laminate thickness, it is presumed that the adjacent cracks are too small to interact with each other. Consequently, only one crack is considered in the half model for the determination of ERR. This is described in detail in reference Burhan et al. (2024a). 3D-Virtual Crack Closure Technique (3D-VCCT) in $${\text{Abaqus}}^{{{\textregistered }}}$$ is used to calculate the ERR along the crack front. Similar to the stress calculation, the 3D model used for computing the ERR employs 8-node linear brick elements (C3D8R) with reduced integration.

**Fig. 6:**
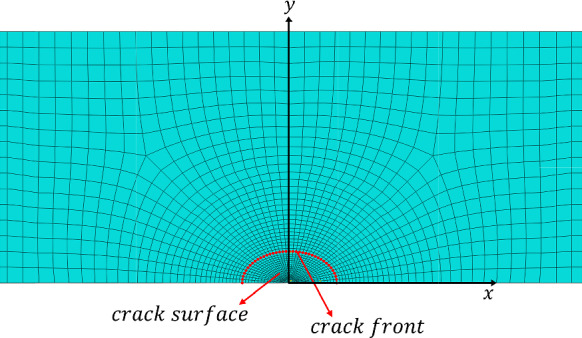
Illustration of a typical FE mesh near semi-elliptical crack front with $\alpha = 3$ and $\beta = 2$ in a cracked laminate

Mesh generation around semi-elliptical cracks, where one axis is significantly larger than the other, introduces complexities. Specifically, creating a mesh for a range of cracks with varying $a$ and $b$ values, especially close to poles aligned with the semi-major axis, poses challenges. To mitigate these issues, a fine mesh is strategically applied around the areas of these poles. The relative crack closure length, i.e., ratio of element size to crack perimeter, at the crack front is maintained as 0.02 to ensure convergence (Burhan et al. 2024c). A typical mesh in the vicinity of a semi-elliptical crack at the free edge with $\alpha = 3$ and $\beta = 2$ is shown in Fig. 6.

## Results and discussion

In the following section, the FE model (interlaminar stresses) and dimensional analysis is validated. Following this, results of interlaminar stresses and IERR, necessary for evaluating the FFM criterion, are presented. The material system under consideration is the carbon epoxy T800/914 laminate. Subsequently, a brief discussion is provided on the material intrinsic properties, including interlaminar fracture toughness and strength. Finally, the proposed 3D FFM prediction of failure thermal load and associated finite crack size for the T800/914 laminate across various ply thicknesses and orientations are discussed. The results are compared against the predictions from TCD (implemented as a second failure criterion) and with both the Cohesive Zone Model (CZM) and 2D FFM from Frey et al. (2021b).

### Validation

The validation of the results is conducted in two steps. First, the FE model for the computation interlaminar stresses, discussed in Sect.  2.5, is validated. Second, the validation of dimensional analysis, presented in Sect. 2.1, is demonstrated.

**Fig. 7:**
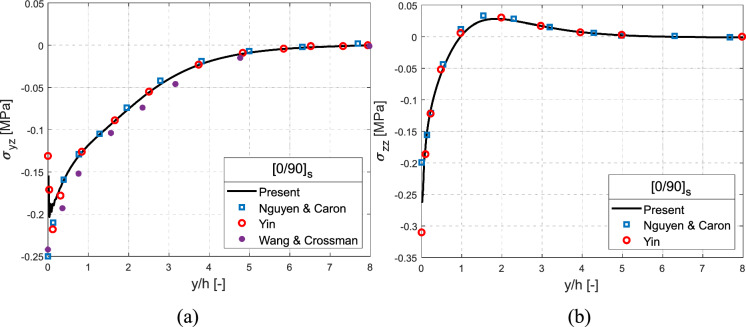
Interlaminar **a** shear stress $\sigma_{yz}$ in comparison with Nguyen and Caron (2009), Yin (1997) and Wang and Crossman (1977) and **b** normal stress $\sigma_{zz}$ in comparison against Nguyen and Caron (2009) and Yin (1997), for the $\left[ {0/90} \right]_{s}$ cross-ply laminate at the ($0/90$) interface under $\Delta T = 1\,^{\circ } {\text{C}}$ uniform thermal load

Figure 7 illustates the interlaminar stress distributions, evaulated using present FE model, at the $0/90$ interface in $\left[ {0/90} \right]_{{\text{s}}}$ laminate subjected to uniform thermal load $\left( {\Delta T = 1\;\,^{\circ } {\text{C}}} \right)$ and is compared with the results of Nguyen and Caron (2009), Yin (1997) and Wang and Crossman (1977). The elastic properties and thermal expansion coefficients utilised are taken from Nguyen and Caron (2009) and are presented in Table 1. Evidently, good agreement between the current FE model and the reference considered is obtained. For a temperature variation of $\Delta T = 1\;\,^{\circ } {\text{C}}$, both the interlaminar shear stress $\sigma_{yz}$ and normal stress $\sigma_{zz}$ are compressive, as presented in Fig. 7a, b, respectively.

**Table 1:** Ply properties of graphite-epoxy unidirectional laminate (Nguyen and Caron 2009)

$E_{1}$(GPa)	$E_{2} = E_{3}$(GPa)	$G_{12} = G_{13} = G_{23}$(GPa)	$\nu_{12} = \nu_{13} = \nu_{23}$	$\alpha_{1}$($^\circ {\text{C}}^{ - 1}$)	$\alpha_{2} = \alpha_{3}$($^\circ {\text{C}}^{ - 1}$)
$137.9$	$14.48$	$5.86$	$0.21$	$0.36 \times 10^{ - 6}$	$28.8 \times 10^{ - 6}$

**Fig. 8:**
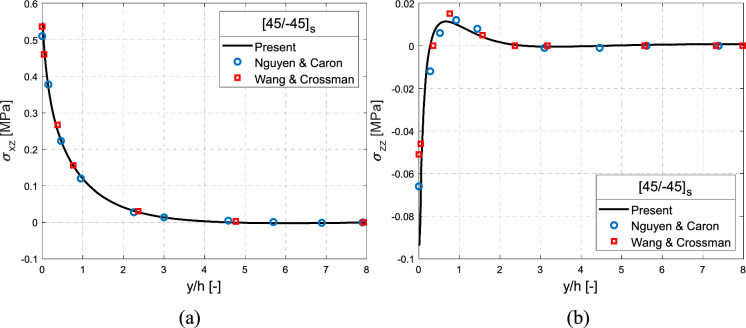
Interlaminar **a** shear stress in comparison against Nguyen and Caron (2009) and Wang and Crossman (1977) and **b** normal stress in comparison against Nguyen and Caron (2009) and Wang and Crossman (1977), for the $\left[ {45/ - 45} \right]_{s}$ angle-ply laminate along the ($45/ - 45$) interface under $\Delta T = 1\,^{\circ } {\text{C}}$ uniform thermal load

In another example of the same material system as above but using angle-ply laminate with $\left[ {45/ - 45} \right]_{{\text{s}}}$ subject to $\Delta T = 1\;\,^{\circ } {\text{C}}$ uniform thermal load is considered. The interlaminar stress distributions are evaulated at the $45/ - 45$ interface and are compared against the results of Nguyen and Caron (2009) and Wang and Crossman (1977), as shown in Fig. 8. The current results align well with the references. The shear stress $\sigma_{xz}$ distribution (Fig. 8a) exhibits positive singularity while normal distribution $\sigma_{zz}$ (Fig. 8b) show negative singularity. Furthermore, it is observed that $\sigma_{xz}$ distribution is prominent in magnitude than $\sigma_{zz}$ distribution and thus delamination is primarily initiated by $\sigma_{xz}$*.* This is consistent with the observations of Frey et al. (2021b) and therefore interlaminar normal stress is not considered in the following considerations.

**Fig. 9:**
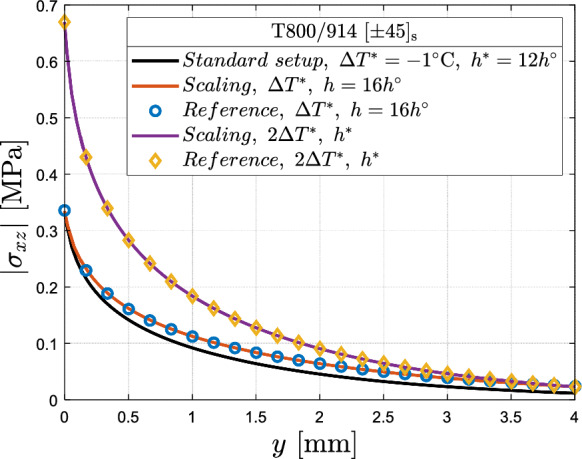
Validation of dimensional analysis using T800/914 laminate with $\left[ { \pm 45} \right]_{s}$ configuration: interlaminar shear stress $\sigma_{xz}$ distribution commencing from a standard laminate configuration (black line) with ply thickness $h^{*} = 12h^{o}$ and thermal load $\Delta T^{*} = - 1\,^{\circ } {\text{C}}$. The dimensional analysis provides $\sigma_{xz}$ distribution for setups of laminate with arbitrary ply thickness and thermal load (coloured lines) and is compared with the reference numerical solutions (markers)

**Fig. 10:**
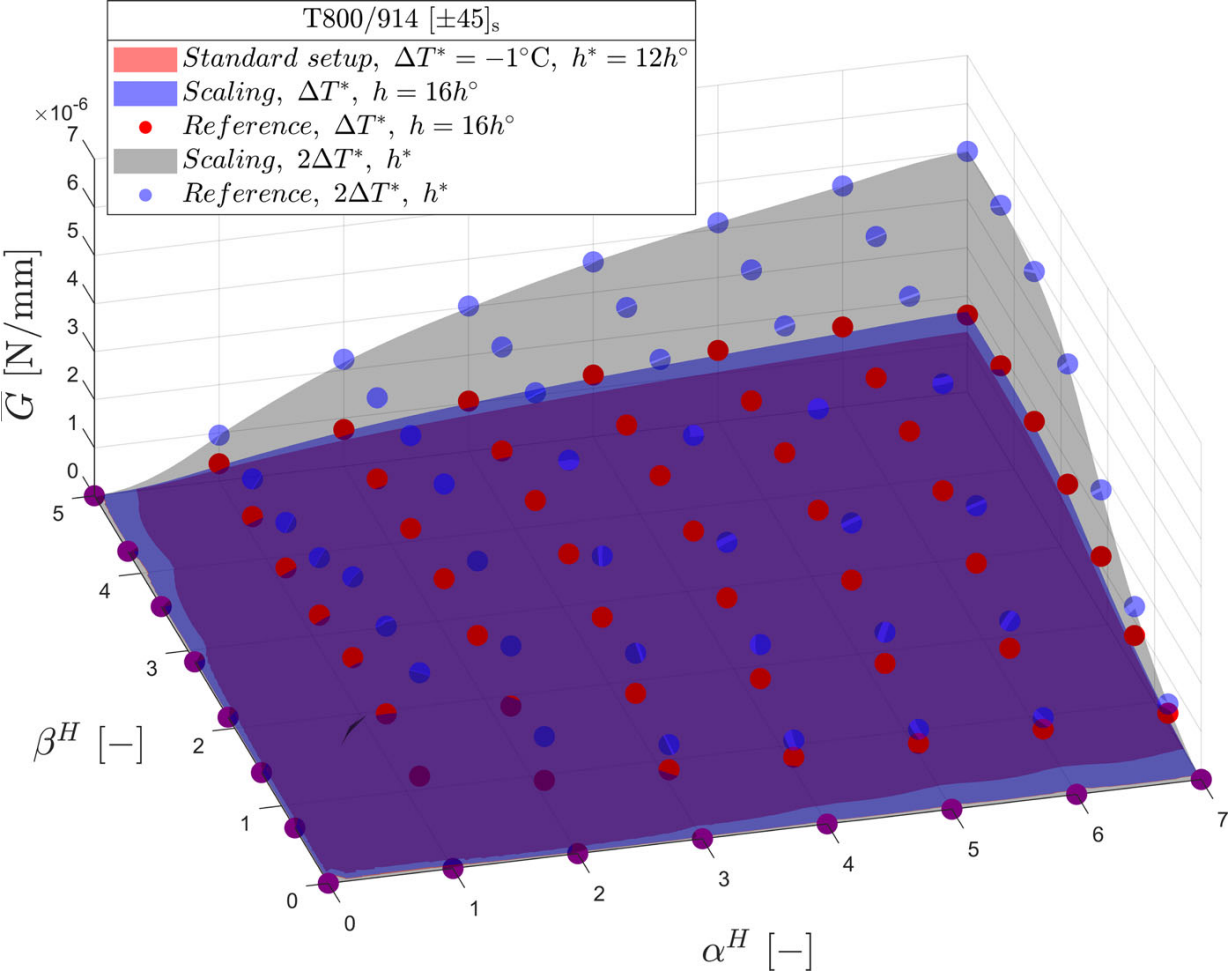
Validation of dimensional analysis using T800/914 laminate with $\left[ { \pm 45} \right]_{s}$ configuration: incremental energy release rate $\overline{G}$ commencing from a standard laminate configuration (red surface) with ply thickness $h^{*} = 12h^{o}$ and thermal load $\Delta T^{*} = - 1\,^{\circ } {\text{C}}$. The dimensional analysis provides $\overline{G}$ as a function of homothetic crack parameters $\alpha^{H}$ and $\beta^{H}$ for setups of laminate with arbitrary chosen ply thickness and thermal load (blue and black surface) and is compared with the reference numerical solutions (markers)

The following investigations involve $\left[ { \pm \theta_{{\text{n}}} } \right]_{{\text{s}}}$ angle-ply laminates composed of T800/914 material. The elastic properties are taken from Leguillon et al. (2001) and thermal expansion coefficients from Lorriot et al. (2003). These properties along with the trace $\Im$ and nominal ply thickness $h^{o}$ are listed in Table 2. As discussed in Sect. 2.1, dimensional analysis eliminates the necessity to re-solve the underlying boundary value problem for different ply thickness and thermal loads. The normalised interlaminar stresses and IERR are semi-analytically evaluated using the FE models (Sect. 2.5) and Eqs. (3), (6), (14). FE models provide first interlaminar stresses and IERR for a given arbitrary load and equations are utilised to evaluate their corresponding non-dimensional functions by inserting the values of these numerically calculated quantities. Subsequently, these normalised functions allow for scaling stresses and IERR to any arbitrary ply thickness and thermal load. Commencing from the standard setup of $\left[ { \pm 45} \right]_{{\text{s}}}$ laminate with $h^{*} = 12h^{o}$ ply thickness subjected to $\Delta T^{*} = - 1\;\,^{\circ } {\text{C}}$ temperature variation. Along the 45/-45 interface, the interlaminar shear stress $\sigma_{xz}$ distribution (see Fig. 9) and IERR $\overline{G}$ (see Fig. 10) are calculated for a laminate under arbitrarily chosen ply thickness and thermal load conditions, distinct from the standard setup. The scaled dimensional analysis solution shows excellent agreement against the reference FE solution for both interlaminar stress and IERR. It is noted here that $\overline{G}$ is plotted as a function of homothetic crack parameters $\alpha^{{\text{H}}}$ and $\beta^{{\text{H}}}$ (see Sect. 2.3), i.e., employing a homothetic coordinate system. It is defined as a coordinate system in which each point represents a potential extension of the semi-elliptical crack along a homothetic path, i.e., maintaining constant crack aspect ratio (Burhan et al. 2024a).

**Table 2:** T800/914 unidirectional elastic properties taken from Leguillon et al. (2001) and thermal expansion coefficients from Lorriot et al. (2003)

$E_{1}$(GPa)	$E_{2}$ = $E_{3}$ (GPa)	$G_{12}$ = $G_{13}$ (GPa)	$G_{23}$(GPa)	$\nu_{12}$ = $\nu_{13}$	$\nu_{23}$	Trace $\Im$ (GPa)	$h^{o}$(mm)	$\alpha_{11}$ ($^\circ {\text{C}}^{ - 1}$)	$\alpha_{22} = \alpha_{33}$ ($^\circ {\text{C}}^{ - 1}$)
$140.23$	$9.57$	$4.85$	$3.24$	$0.35$	$0.48$	$196$	$0.125$	$0.02 \times 10^{ - 6}$	$22.5 \times 10^{ - 6}$

### Interlaminar stresses and incremental energy release rates

**Fig. 11:**
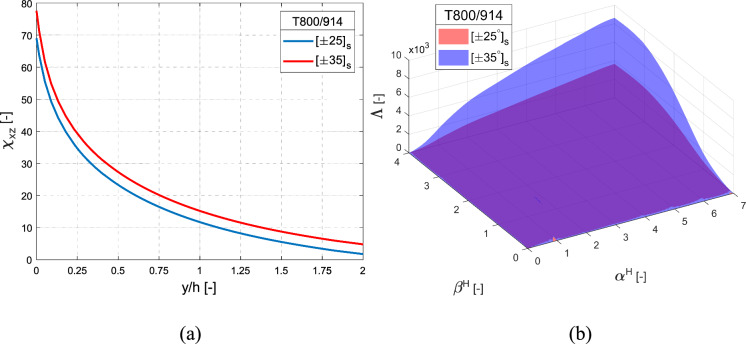
Normalised **a** interlaminar shear stress $\chi_{xz}$ and **b** incremental energy release rate $\Lambda$ as a function of homothetic crack parameters $\alpha^{H}$ and $\beta^{H}$, for angle-ply laminate along the ($\theta / - \theta$) interface

Interlaminar stresses and IERR are important input quantities for the FFM. This section discusses these quantities for the $\left[ { \pm \theta } \right]_{{\text{s}}}$ angle-ply laminates at the dissimilar $\theta / - \theta$ interface under remote thermal loading. For the material considered with selected ply orientation, Fig. 11a, b illustrates the normalised shear stress distribution $\chi_{xz}$ and IERR ${\Lambda }$, respectively. Both non-dimensional functions increase with increase in ply orientation. The distribution of $\chi_{xz}$ exhibits a singularity at the free edge, which rapidly diminishes towards the interior of the laminate. Furthermore, it is observed from the strength of the materials perspective, for the considered ply orientations*,* higher angles of laminates are more susceptible to initiate delamination. The normalised IERR ${\Lambda }$, as a function of homothetic crack parameters $\alpha^{{\text{H}}}$ and $\beta^{{\text{H}}}$, increases with $\alpha^{{\text{H}}}$, however decreases slightly at higher values of $\beta^{{\text{H}}}$.

### Interface fracture properties

The current 3D FFM fracture criterion for angle-ply laminates subject to thermal load that induces free edge delamination requires two material intrinsic properties, i.e. interfacial fracture toughness $G_{c}$ and shear strength $S_{x}$. In general, these interface properties are unknown since they depend upon fibre, matrix and ply orientation (Donaldson 1988; Liao and Sun 1996; Andersons and König 2004). Furthermore, the influence of size effects on material interface properties is often observed. Studies by Laffan et al. (2010), Harris and Morris (1984), and Donaldson (1988) have all reported a reduction in fracture toughness as ply thickness increases. On the other hand, Diaz and Caron (2006) argue that interlaminar strength varies with the model layer thickness and should not be regarded as an intrinsic material property. Size effects on the measured interlaminar shear strength are widely recognised (Wisnom 1999), especially in cases where matrix failure predominates.

Consequently, material parameters are often determined by inverse fits. Lorriot et al. (2003) and Lagunegrand et al. (2006) used least square method for assessment of both critical distance and interlaminar strength by minimising deviation between their average stress criterion model and experimental results. Martin et al. (2010) and Dölling et al. (2020), applied the root-mean-square approach to determine the optimum couple ($G_{c}$, $S_{x}$) by minimising the deviation between their FFM model and experimental data. Martin et al. (2010) employed this approach to identify the couple that satisfies the condition where the relative error falls within the relative experimental deviation (derived from experimental scattering), whereas Dölling et al. (2020) identified the best couple for a given interlaminar shear strength.

In another study Burhan et al. (2024a), the couple $$(G_{c}$$, $$S_{x} )$$ is identified by using a root-mean-square fit to minimise deviations in both critical load and predicted average delamination onset length between their 3D FFM model and experimental results. The deviation in average delamination initiation is minimised against the critical values identified from the experiments (Lagunegrand et al. 2006; Lorriot et al. 2003; Brewer and Lagace 1988). One of the material system in this study considered is T800/914 and the mixed/mode fracture toughness for angle-ply laminates with $${\uptheta }$$ = $$10\,^{\circ }$$, $$20\,^{\circ }$$ and $$30\,^{\circ }$$ is also determined. In the present study, this data of toughness is adopted and are listed in the Table 3 along with the interlaminar shear strength taken from Gu and Huang (2019). The toughness illustrates the decreasing trend with increase in ply orientation angle. This observation is consistent with reference Donaldson (1988). Furthermore, the interface toughness values required in the current study within inside/outside these ply orientations ($${\uptheta }$$ = $$10\,^{\circ }$$, $$20\,^{\circ }$$, $$30\,^{\circ }$$) are assessed using linear interpolation and extrapolation.

**Table 3:** Interfacial fracture parameters ($G_{c}$, $S_{x}$) for T800/914 laminate

Laminate	$10\,^{\circ }$	$20\,^{\circ }$	$30\,^{\circ }$	$S_{x}$(MPa)
	$$G_{c}$$(N/mm)			
T800/914	0.2	0.1	0.08	70

Fracture toughness taken from Burhan et al. (2024a) and interlaminar shear strength from Gu and Huang (2019)

### Prediction of critical temperature loads

**Fig. 12:**
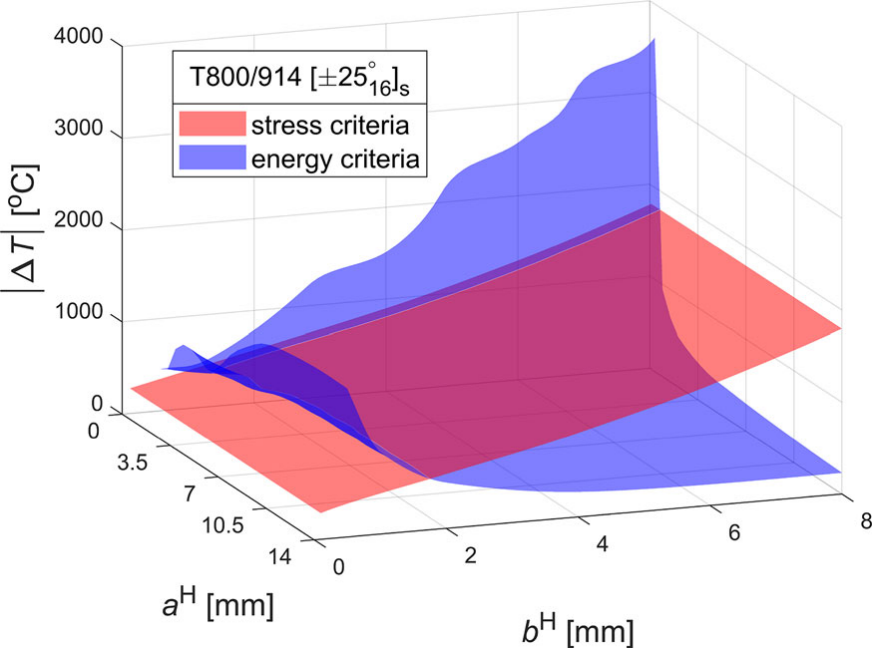
Illustration of stress and energy criteria for 3D FFM thermal analysis: the necessary $\left| {\Delta T} \right|$ values for delamination onset at the 25/-25 interface of the T800/914 laminate with stacking sequence $\left[ { \pm 25_{16} } \right]_{s}$ are depicted as a function of homothetic crack lengths $a^{\rm{H}}$ and $b^{\rm{H}}$

This section outlines the proposed 3D FFM criterion for predicting critical thermal loads for angle ply $\left[ { \pm \theta_{{\text{n}}} } \right]_{{\text{s}}}$ laminates composed of the carbon/epoxy T800/914 system. It compares these predictions with results obtained from TCD, and with both the CZM and the 2D FFM from Frey et al. (2021b), all of which are considered reference models. These predictions are made using the material parameters listed in Table 3. Notably, the same value of interlaminar strength (see Table 3) is applied in the prediction of critical thermal loads using TCD. The inequalities presented in Eq. (16) provide the failure thermal load for crack nucleation as a function of homothetic crack lengths $a^{{\text{H}}}$ and $b^{{\text{H}}}$, based on stress and energy conditions, respectively. These evaluations are illustrated graphically for the ply $\left[ { \pm 25_{16} } \right]_{{\text{s}}}$ laminate in Fig. 12. The two surfaces in the graph represent the determined temperature difference $\Delta T$ according to two conditions. Interestingly, according to stress condition, smaller $\Delta T$ values are required to initiate cracks of smaller delamination width $b$. Conversely, for larger values of $a$ and $b$, smaller $\Delta T$ is required to initiate crack as the per energy condition. Furthermore, the intersection curve of the two surfaces represents infinite solutions that exist for the FFM system (16). Along this intersection curve, a region corresponding to minimum $\Delta T$ values can be observed, where similar values of $\Delta T$ and $b$ exist. This indicates a spontaneous crack extension in $b$ direction, potentially leading to longer and shallow cracks. The unique solution of Eqs. (16) is obtained by employing a standard optimisation algorithm, as discussed in Sect. 2.3. The constrained standard nonlinear optimisation problem is solved using the MATLAB function *fmincon*.

**Fig. 13:**
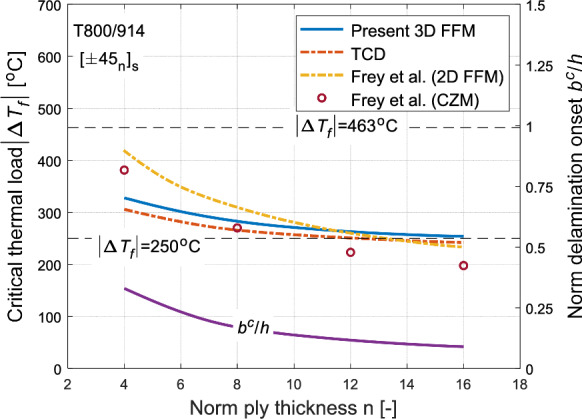
Prediction of failure thermal load $\left| {\Delta T_{f} } \right|$ of T800/914 laminate with stacking sequence $\left[ { \pm 45_{{\text{n}}} } \right]_{{\text{s}}}$, using the current 3D FFM (solid blue line), compared against predictions from TCD (red dashed line) and of those by Frey et al. (2021b) (yellow dashed line for 2D FFM and red circle for CZM), with respect to normalised ply thickness n. The corresponding determined normalised delamination initiation width $b^{c} /h$ (solid purple line) is shown right y-axis

The first prediction focuses on the $\left[ { \pm 45_{{\text{n}}} } \right]_{{\text{s}}}$ laminate with respect to varying normalised ply thickness n. Figure 13 illustrates the critical thermal loads $\Delta T_{f}$ predicted by the current 3D FFM model and compares it with the reference models. Overall, good agreement is observed among all four models, although the 3D FFM and TCD results tend to be slightly conservative than CZM for smaller ply thicknesses. Additionally, at these thicknesses, 3D FFM predictions fall between the reference models. The failure thermal load $\Delta T_{f}$ is noted to decrease with an increase in ply thickness, indicating size effects. This phenomenon can be attributed to the fact that thicker laminates store more energy, which then is available for crack formation. The ability of 3D FFM to adequately replicate these size effects is due to its consideration of energy balance. Furthermore, size effects have been reported in many experimental studies Lagunegrand et al. (2006), Brewer and Lagace (1988), Lorriot et al. (2003**),** Diaz and Caron (2006). The normalised delamination onset width $b^{c} /h$ determined from 3D FFM is observed to decrease with increasing ply thickness and is presented on the right $y$-axis in Fig. 13.

**Fig. 14:**
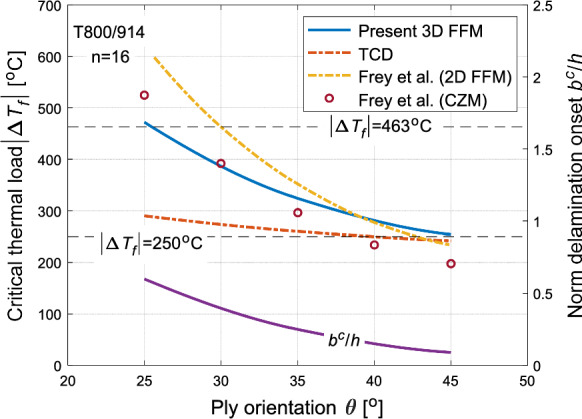
Prediction of failure thermal load $\left| {\Delta T_{f} } \right|$ of T800/914 laminate with $\left[ { \pm \theta_{16} } \right]_{{\text{s}}}$ configuration, using the current 3D FFM (solid blue line), compared to predictions from TCD (red dashed line) and those by Frey et al. (2021b) (yellow dashed line for 2D FFM and red circle for CZM), with respect to ply orientation $\theta$. The corresponding determined normalised delamination initiation width $b^{c} /h$ (solid purple line) is shown right y-axis

For a given normalised ply thickness n = 16, the prediction of critical thermal load $\Delta T_{f}$ for $\left[ { \pm \theta_{16} } \right]_{{\text{s}}}$ laminates are presented and discussed with respect to varying ply orientation. Figure 14 illustrates the current 3D FFM predictions and compares them with predictions from reference models Additionally, the determined normalised delamination onset width $b^{c} /h$ using 3D FFM is plotted on the right $y$-axis. Generally, close agreement is obtained between current 3D FFM predictions and those of Frey et al. (CZM and 2D FFM), except for slightly conservative results predicted by 3D FFM at lower ply orientation angles. Conversely, TCD predictions are significantly conservative at lower ply orientation angles, with 3D FFM predictions falling between the reference models. The deviation between current 3D FFM model and the 2D FFM model by Frey et al. may stem from two reasons. Firstly, the choice of interlaminar strength in the present study, which influence the predictions, as reported in many studies (Martin et al. 2010; Burhan et al. 2024a). Secondly, Frey et al. have implemented a point stress criterion in their 2D FFM model, while current 3D FFM utilises an average stress criterion. Additionally, Frey et al. compared their 2D FFM predictions against CZM. The predictions of CZM also yielded conservative results with a relative error of 19%. Interestingly, the maximum relative error between current 3D FFM and that 2D FFM of Frey et al. is closer, around 24%. Although current 3D FFM predictions are acceptable, better agreement could be achieved either by utilising an appropriate value for interlaminar strength in the present model or by implementing an average stress criterion in their model. Moreover, TCD predictions being conservative may be due to the values utilised for critical length and interlaminar strength. Since a constant interlaminar strength value is used for both TCD and 3D FFM in the current study, selecting an appropriate pair of values for interlaminar strength and critical length in the TCD model may reduce the deviation between TCD and other models. It is noted here that although the delamination initiation of angle-ply laminates considered in the current study is for pure thermal loading, the effects of temperature change become imperative also when laminates are subjected to thermo-mechanical loading. In such cases, laminate strength may be significantly reduced by temperature variation prior to any mechanical loading, owing to the pre-stressed condition of laminates (Frey et al. 2021b).

Frey et al. (2021b) further elaborated on the significance of the temperature at crack initiation $T_{f}$. The FFM fracture criterion yields the modulus of temperature change $\left| {\Delta T_{f} } \right|$. This $\Delta T_{f}$ is expressed as $T_{f} - T_{o}$, where $T_{o}$ represents the stress-free temperature of the considered matrix. For 917 epoxy $T_{o}$ is equal to $190\,^{\circ } {\text{C}}$ according to the manufacturer. Notably, the glass-transitioning temperature also coincides with $190\,^{\circ } {\text{C}}$ and therefore temperature beyond this threshold value is not relevant. This is due to the reason that the properties of epoxy change rapidly beyond this temperature, making $190\,^{\circ } {\text{C}}$ an upper limit of considered temperature range. Moreover, absolute zero temperature ($T_{f} = - 273\,^{\circ } {\text{C}}$) can be regarded as lower limit of this range. Consequently, the considered failure temperature change range spans $- 463\,^{\circ } {\text{C}} \le \Delta T_{f} \le 0\,^{\circ } {\text{C}}$. For most technical applications within the atmospheric conditions, temperatures can descend to $- 60\,^{\circ } {\text{C}}$, making maximum value of $\left| {\Delta T_{f} } \right| = 250\,^{\circ } {\text{C}}$. However, for space applications, $\left| {\Delta T_{f} } \right| \ge 250\,^{\circ } {\text{C}}$. These temperature limits are shown in both Figs. 13 and 14. It can be inferred that laminates with lower ply orientation angles and thicknesses are highly unlikely to experience delaminate within the considered temperature change range. Therefore, Frey et al. suggested designing laminates with numerous thin plies of alternating angles rather than fewer thick ones. The current 3D FFM results align with their findings, indicating that both provide similar insights into laminate design. Specifically, laminates with thicker plies and higher ply orientations are more susceptible to delamination initiation compared to laminates with thinner and lower orientation plies within the considered temperature change range.

## Concluding remarks

This study investigates the free edge delamination in angle-ply laminates subjected to thermal loading using the 3D FFM fracture criterion. Essential input quantities such as interlaminar stresses and incremental energy release rates required for FFM are determined semi-analytically through the implementation of FE models and dimensional analysis. The expressions for non-dimensionalised functions derived from dimensional analysis, corresponding to these two input quantities, are independent of thermal load and ply thickness. Consequently, the current framework eliminates the necessity of re-solving the underlying boundary value problem for an arbitrary thermal load or ply thickness. The FE model utilised for determining the distribution of interlaminar stresses is validated through two test cases (cross and angle-ply laminates) against numerical results from the literature. Furthermore, dimensional analysis is validated against reference finite element solutions. Excellent agreement is obtained in both validations. The 3D FFM system is solved by employing a standard constrained nonlinear optimisation problem by assuming a homothetic crack extension. A reference model based on the Theory of Critical Distances is employed, along with both the CZM and 2D FFM reference models by Frey et al. sourced from the literature, to compare the predictions of failure thermal loads by the current 3D FFM. Good agreement is obtained between the current 3D FFM predictions and those in the literature. However, for a given pair of values of interlaminar strength and critical length, the Theory of Critical Distances model tends to provide significantly conservative predictions of failure thermal loads.

## Data Availability

Data will be made available on request.

## Nomenclatures

$a$, $b$: Two semi-axes of a semi-elliptical crack
$E_{1} ,E_{2} , G_{12} ,\upsilon_{12} ,\upsilon_{23}$: Elastic properties of the ply
$E$, $\upsilon$: Elastic properties of a resin-rich layer
$G$: Energy release rate
$\overline{G}$: Incremental energy release rate
$G_{c}$: Interfacial fracture toughness
$$h$$: Ply thickness
$h^{o}$: Nominal ply thickness
$I/II/III$: Different modes of fracture
$L$: Length of laminate
n: Normalised ply thickness $h/h^{o}$
$S_{x}$, $S_{y}$, $S_{z}$: Interlaminar strengths for $\sigma_{xz} ,$ $\sigma_{yz}$, $\sigma_{zz}$
$t$: Total thickness of the laminate
$W$: Half-width of laminate
$x, y, z$: Global coordinate system
$$\left( \cdot \right)^{c}$$: Critical value
$\left( \cdot \right)^{{\text{H}}}$: Homothetic coordinate system
$\Im$: Trace of a material
$\alpha_{1} , \alpha_{2}$: Thermal coefficients of the ply
$\alpha_{p}$: Thermal expansion coefficient of resin-rich layer
$$\alpha ,\beta$$: Crack parameters
$\Delta T$: Uniform temperature change
$\Delta T_{f}$: Critical thermal load
$$\theta$$: Ply orientation
${\Lambda }$: Normalised incremental energy release rate
$$\sigma_{xz} ,$$$$\sigma_{yz}$$, $$\sigma_{zz}$$: Interlaminar stress components
$\phi$: Polar angle at the semi-elliptical crack front
$$\chi ,$$$$\psi$$: Stress function and correction factor
CFRP: Carbon Fibre-Reinforced Polymer
CZM: Cohesive zone model
ERR: Energy release rate
FEM: Finite element method
FFM: Finite fracture mechanics
IERR: Incremental energy release rate
VCCT: Virtual crack closure technique
